# Viral RNA persistence and long-term consequences following acute infection with Risk Group 4 zoonotic viruses

**DOI:** 10.3389/fcimb.2026.1809435

**Published:** 2026-04-29

**Authors:** Hongzhao Li, Bradley S Pickering

**Affiliations:** 1 National Centre for Foreign Animal Disease, Canadian Food Inspection Agency, Winnipeg, MB, Canada; 2 Department of Medical Microbiology and Infectious Diseases, College of Medicine, Faculty of Health Sciences, University of Manitoba, Winnipeg, MB, Canada

**Keywords:** arenavirus, bunyavirus, filovirus, flavivirus, henipavirus, risk group 4, the latency hypothesis, viral RNA persistence

## Abstract

RNA viruses are historically characterized by acute, strictly transient infections, with few exceptions. However, the emerging phenomenon of viral RNA persistence is fundamentally challenging this paradigm. Accumulating data across non-retroviral RNA viruses including several families of Risk Group 4 (RG4) pathogens demonstrate that viral genetic material can persist within the host long after clinical recovery and the clearance of systemic viremia. The current review explores this new frontier in infectious diseases, synthesizing recent evidence for viral RNA persistence in RG4 zoonotic viruses and its profound clinical and public health implications, from post-acute sequelae and fatal recrudescence to the risk of re-igniting outbreaks. We further attempt to integrate findings from disease-susceptible hosts with those from natural reservoir hosts and propose a hypothetical, central role of viral RNA persistence in the maintenance and transmission of zoonotic RNA viruses, an idea we refer to as “the Latency Hypothesis”. These updates underscore the critical need to elucidate the molecular mechanisms underlying persistence and to develop targeted medical countermeasures.

## 1 Introduction

Traditionally, RNA viruses were believed to cause exclusively acute infections due to the inherent instability of RNA and the absence of recognized latency programs analogous to those of many DNA viruses. As pointed out by Diane E. Griffin, a major founding pioneer in the study of viral RNA persistence, however, accumulating evidence contradicts this assumption, revealing that RNA from diverse viral families can persist long after host recovery from acute infections, for many weeks, months or years (Griffin, 2022).

It is important to clarify terms: Persistent viral RNA, as detected by molecular assays (such as RT-PCR and *in situ* hybridization), includes viral genomic or subgenomic sequences that can be in single-stranded or double-stranded forms and may occur at sanctuary sites such as the reproductive and nervous systems and lymphoid organs. “Persistence” here typically refers to the continued detection of viral RNA, not necessarily ongoing productive infection or shedding of infectious virions. Many studies fail to recover culturable virus but do detect viral RNA. However, the potential of reactivation from persisting viral RNA to reproduce infectious virus, which might occur in an intermittent manner and within short time windows, remains to be understood in general. Moreover, persistence may be asymptomatic, or may correlate with prolonged, relapsing or late‐onset symptoms (Griffin, 2022).

**Figure 1:**
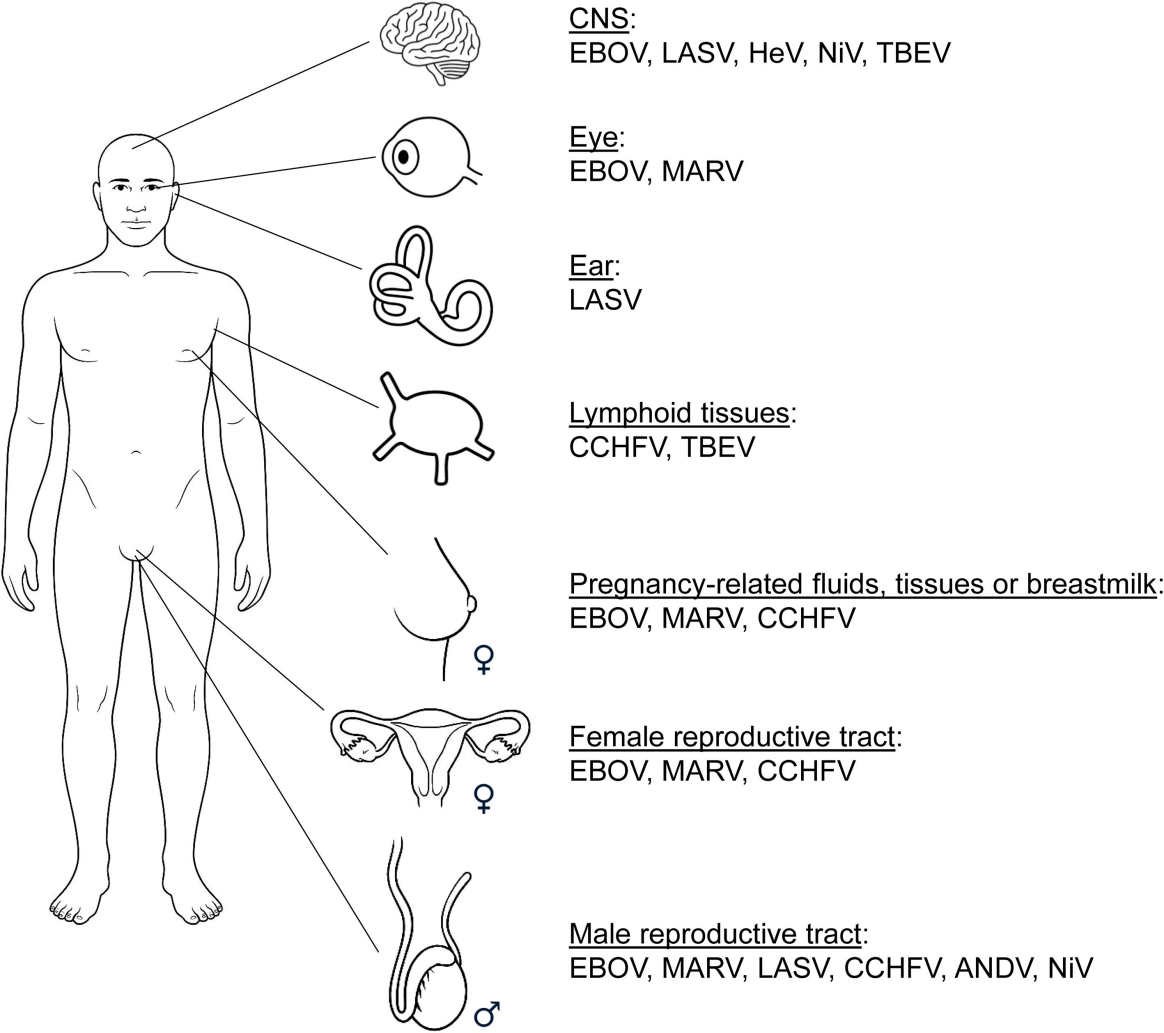
Persistence of RG4 viral RNA in tissues. CNS, central nervous system; EBOV, Ebola virus; LASV, Lassa virus; HeV, Hendra virus; NiV, Nipah virus; TBEV, tick-borne encephalitis virus; MARV, Marburg virus; CCHFV, Crimean-Congo hemorrhagic fever virus; ANDV, Andes virus. Anatomical compartments harboring persistent viral RNA are illustrated using a schematic format consistent with previously published reviews (Griffin, 2022; Meek et al., 2025).

This review focuses on non-retroviral, non-integrating RNA viruses, excluding those with established chronic replication cycles such as human immunodeficiency virus and hepatitis C virus (Griffin, 2022) and is specifically dedicated to Risk Group 4 (RG4) zoonotic viruses. RG4 represents the highest level of biological hazard, primarily distinguished from lower groups by the combination of extreme disease severity, high transmissibility and a lack of effective medical countermeasures. Viral RNA persistence has been observed across several families of RG4 viruses, including those prioritized by the World Health Organization for research and development in emergency contexts, Ebola (EBOV), Marburg (MARV), Lassa (LASV), Crimean-Congo hemorrhagic fever (CCHFV), Hendra (HeV) and Nipah (NiV) viruses (WHO, 2026). Two additional RG4 viruses reviewed here are Andes virus (ANDV) and tick-borne encephalitis virus (TBEV). We synthesize recent data on the detection of persistent viral RNA and related clinical consequences and risks to public health for each of these viruses (summarized in **Figure 1** and **Supplementary Table 1** and elaborated below). While the biology of viral RNA persistence remains poorly understood, we also offer mechanistic perspectives that we hope will inspire future investigations.

## 2 EBOV

### 2.1 Introduction to EBOV

The genus *Orthoebolavirus* (formally *Ebolavirus*), within the family *Filoviridae* (characterized by a non-segmented, negative-sense, single-stranded RNA genome), comprises six species: Ebola (Zaire), Sudan, Bundibugyo, Taï Forest, Reston and Bombali viruses (Cheng et al., 2025). The first three are responsible for outbreaks of severe diseases in humans. For simplicity, in the context of this review, “EBOV” refers to the Zaire species and serves as a general term that represents the genus unless otherwise specified. EBOV is the prototype virus in the genus that demonstrates the highest levels of disease burden, research breadth and depth, and scientific advancement, including in the field of viral RNA persistence.

EBOV causes endemic and epidemic outbreaks primarily in Central and West Africa (CDC, 2026). It is widely accepted that EBOV infection is a zoonosis (an infectious disease caused by an agent transmitted between animals and humans). Retrospective epidemiological studies often succeed in tracing the likely index cases. These individuals are commonly linked to contact with wildlife or their carcasses (Judson et al., 2016). The reservoir host(s) of EBOV currently remain uncertain. Bats have been implicated as the most plausible reservoirs (Leroy et al., 2005; Riesle-Sbarbaro et al., 2024), based on the successful isolation of MARV, a related filovirus, from Egyptian rousette bats (Rousettus aegyptiacus) (Towner et al., 2009; Amman et al., 2012) and detection of small fragments of EBOV RNA and EBOV antibodies from three bat species (*Hypsignathus monstrosus*, *Epomops franqueti* and *Myonycteris torquata*) (Leroy et al., 2005). EBOV RNA fragments or antibodies were also found in several mammalian species other than bats, including nonhuman primates (NHPs) (Morvan et al., 1999; Leroy et al., 2004a, 2004; Wittmann et al., 2007), suggesting that the reservoir may possibly involve more than a single animal species or group. The specific role of each species in sustaining the long-term reservoir is unknown. Despite extensive sampling efforts, however, no live virus has been isolated from any naturally infected hosts (McCrone et al., 2025). It is implied that live virus encounter is a rare event and episodic re-emergence of spillovers is regulated by specific conditions, which remain elusive.

### 2.2 Ebola virus disease

EBOV transmission to humans is believed to occur through contact with infected wildlife, followed by sustained human-to-human spread via direct exposure to blood, bodily fluids and contaminated materials, including during caregiving and burial practices (Rewar and Mirdha, 2014). In acute EVD, a sudden onset of non-specific, influenza-like symptoms is followed by multi-system involvement with gastrointestinal, respiratory, vascular and neurological manifestations. In fatal cases, patients die with hypovolemic shock and multi-organ failure, typically between days 6 and 16 after symptom onset. Case fatality rates are around the range of 60–90%. In non-fatal cases, patients improve and recover typically from days 6–11, which coincides with antibody development (Feldmann and Geisbert, 2011).

### 2.3 EBOV viral RNA persistence and long-term impacts on patient and public health

The 2014–2016 West African epidemic (28,610 total cases, 11,308 deaths and 17,302 survivors) and 2018–2020 Democratic Republic of the Congo (DRC) epidemic (3,470 total cases, 2,287 deaths and 1,183 survivors), along with numerous other outbreaks (CDC, 2026), have provided unprecedented numbers of survivors of acute infections and a unique opportunity to reveal the previously underappreciated importance of viral RNA persistence. In patients who survive an acute case of EVD, despite the clearance of viremia, development of immune responses and apparent clinical recovery, it has been extensively observed that viral RNA (and occasionally infectious virus) can remain for months to years in immune-privileged sites and associated bodily fluids.

#### 2.3.1 Semen and the male reproductive tract

The strongest and most extensively documented evidence for EBOV RNA persistence involves the male reproductive tract. Persistent viral RNA in semen has been implicated in numerous new outbreaks.

In earlier case studies, EBOV viral RNA (Rowe et al., 1999; Christie et al., 2015) or infectious virus (Rodriguez et al., 1999) was detected in semen samples of EVD survivors. Extended cohort studies further confirmed the prevalence of long-term persistence of EBOV RNA in semen (Deen et al., 2017; Sissoko et al., 2017a; Keita et al., 2019; Sneller et al., 2019; Kofman et al., 2021; Thorson et al., 2021; Dyal et al., 2023). While the numbers vary among different cohort studies, the detection rates of EBOV RNA in semen typically were 100% within three months after discharge from Ebola treatment units (ETU) and 62–75.4% at 4–6 months post ETU, with a declining trend over time and a median persistence duration of 204 days (Deen et al., 2017; Thorson et al., 2021). The longest times reported for EBOV RNA persistence in semen have been “40 months from acute EVD illness” (Sneller et al., 2019) and 988 days (33 months) following discharge from acute EVD recovery (Kofman et al., 2021).

The prevalence of EBOV RNA persistence in semen raised the concern of potential sexual transmission. Although most studies that detected viral RNA persistence either did not perform or was not successful in the isolation of live virus, viral RNA persistence was indeed linked to a series of re-emerging infection events. A seminal study combining epidemiologic and genomic data provided evidence of sexual transmission from a male survivor to a female patient on day 179 post EVD onset (Mate et al., 2015). This is the first of the three flare-ups reported for Liberia in 2015 (March). EBOV RNA persisted in the semen of the male survivor of this study for at least 199 days after the estimated onset of EVD, or 175 days after the clearance of viremia. Another case study reported EBOV RNA persistence in seminal fluid 531 days and sexual transmission 470 days post symptom onset (Diallo et al., 2016). Similar findings of male-to-female sexual transmissions involving viral RNA persistence in semen were also reported from other flare-ups (Subissi et al., 2018).

#### 2.3.2 Ocular compartment

Early clinical observations showed that the eye is a major site of post-EVD sequelae and recrudescence potentially linked to EBOV RNA persistence. Studies from the 1995 Kikwit outbreak documented the frequent development of uveitis, other ocular manifestations such as ocular pain, photophobia and hyperlacrimation and vision impairment, and the ocular persistence of EBOV RNA (Kibadi et al., 1999; Rodriguez et al., 1999). The significance of this phenomenon was further strengthened during the 2014–2016 West Africa epidemic, when a landmark case study demonstrated viable EBOV in aqueous humor in survivors with severe uveitis 9 weeks after the clearance of viremia (Varkey et al., 2015).

#### 2.3.3 Central nervous system

Persistent EBOV RNA or viral reactivation within the CNS can result in severe or fatal late neurological consequences, as evidenced in three well-substantiated human cases demonstrating disease recrudescence (Jacobs et al., 2016; Mukadi-Bamuleka et al., 2024). It is noted that before their ETU discharge from acute EVD recovery, the first patient received brincidofovir (antiviral drug) and convalescent plasma, followed by an anti-EBOV monoclonal antibody (mAb) therapy (ZMAb, Public Health Agency of Canada). The other two patients were given post-exposure vaccination with rVSV-ZEBOV (Merck) and treatment with REGN-EB3 (EBOV mAb cocktails, Regeneron Pharmaceuticals). These patients relapsed with severe (the first patient) and fatal meningoencephalitis with high-level EBOV RNA in CSF nine months, 342 days (about 11 months) and137 days (about 5 months) post discharge, respectively.

#### 2.3.4 Vaginal fluids

EBOV RNA was found to persist in vaginal fluids of female EVD survivors for at least 33–36 days after disease onset (Rodriguez et al., 1999; Liu et al., 2019c). While these data suggest a theoretical risk of female−to−male sexual transmission or vertical transmission, epidemiological investigations have not linked any outbreak flare−ups to female genital shedding.

#### 2.3.5 Pregnancy-related fluids, tissues and breastmilk

Across multiple outbreaks, EBOV RNA was repeatedly identified in pregnancy-associated tissues and fluids, demonstrating that the maternal–fetal interface and lactational pathways can serve as reservoirs of viral RNA with potential for vertical or peripartum transmission. A stillborn infant tested positive with EBOV RNA and virus (Bower et al., 2016) and stillborn fetuses (EBOV RNA status not determined) (Caluwaerts et al., 2016) were delivered by mothers who had clinically recovered from EBOV infection and had been EBOV RNA-negative in blood. In the latter study, EBOV RNA was detected in vaginal swab (after rupture of amniotic sac), amniotic fluid, placental swab and cord blood (Caluwaerts et al., 2016). Breastmilk was identified as a particularly important site of EBOV RNA persistence. A case study (Sissoko et al., 2017b) reported EBOV RNA in the breast milk of an asymptomatic mother positive for EBOV IgG antibodies whose infant subsequently died of EVD, with genomic analysis strongly suggesting viral transmission through breastfeeding. Longer-term cohort research confirmed that EBOV RNA can persist months after recovery, with documented rare but notable instance of EBOV RNA detection in breast milk up to 500 days post ETU discharge (Keita et al., 2019). Systematic evidence reviews reinforce these findings: EBOV RNA was detected in amniotic fluid up to 32 days after maternal viral clearance from the blood and in breastmilk up to 26 days after symptom onset, suggesting that precautions should be taken concerning the potential risk associated with pregnancy-related fluids beyond maternal recovery (Foeller et al., 2020). Another review (Medina-Rivera et al., 2021) identified EBOV RNA or virus in breastmilk samples from seven out of ten mothers across published case reports, with fatal infections in the majority of the exposed infants, underscoring the biological plausibility of mother-to-child transmission through lactation. A more recent study (Fallah et al., 2023) found that EBOV RNA was not or rarely detected in placental, cord blood, vaginal, or breastmilk samples collected at delivery from people who conceived at 10–20 months (median 14 months) after surviving EVD, suggesting that on these time scales the long-term risk of pregnancy-associated viral transmission is low.

#### 2.3.6 Unidentified tissue sites of persistence linked to late disease recrudescence and transmissions

During the 2018–2020 DRC epidemic, a patient previously vaccinated with rVSV-ZEBOV (Merck) recovered from acute EVD after treatment with mAb114. Five months after his discharge from ETU, the patient developed a severe, fatal case of EVD, with genomic investigations confirming it as a relapse rather than a new infection. This recrudescence triggered an onward transmission chain of 91 new infections and extended the epidemic by four months. The tissue site of viral RNA persistence was unclear while a semen test was negative at two months post discharge (Mbala-Kingebeni et al., 2021).

#### 2.3.7 Potential new tissue sites of persistence

Recent evidence suggests that adipose tissue may represent a new tissue type of EBOV tropism and a previously overlooked reservoir site capable of supporting EBOV RNA persistence, expanding the framework of potential long−term viral sanctuaries beyond classical immune−privileged sites. Earlier investigation in a guinea pig lethal infection model (with a guinea pig-adapted EBOV strain) found an abundance of EBOV RNA in peritoneal fat (Connolly et al., 1999). A subsequent study in a mouse lethal infection model (with a mouse-adapted EBOV strain) demonstrated viral RNA in visceral and subcutaneous adipose tissue and determined that human adipocytes are susceptible to EBOV infection (Gourronc et al., 2022). To assess the potential of longer-term persistence of EBOV RNA in adipose cells, the latest study on this topic reported viral replication for at least 28 days without overt cytopathic effects during *in vitro* infection of human and bat adipose tissue cultures using wild-type EBOV (Garnett et al., 2023). In addition, EBOV infection of adipose tissue was found to have effect on interferon and inflammatory cytokine responses and metabolic functions of adipocytes (Gourronc et al., 2022; Garnett et al., 2023). It remains to be further investigated how the adipose tropism and associated effects may play a role in EBOV persistence in human survivors, maintenance in wildlife reservoirs and the ecological dynamics of outbreak re−emergence. Such studies bear substantial significance as adipose tissue is highly abundant and broadly present across mammalian hosts.

#### 2.3.8 Unidentified primary case with RNA persistence leading to new outbreaks

In several EBOV outbreaks where no primary case could be identified, epidemiological and genomic analyses pointed to a persistently infected source (i.e. survivor of a previous outbreak). The times of EBOV RNA persistence leading up to the new outbreaks can be months or years. These were described for the latter two of the three flare-ups in Liberia in 2015 (June and November) (Blackley et al., 2016; Subissi et al., 2018), and the flare-up in Sierra Leone in 2016 (Alpren et al., 2016; Subissi et al., 2018). Furthermore, the most remarkable lesson was learned from the 2021 Guinea epidemic: A landmark study (Keita et al., 2021) provided clear phylogenetic evidence that viral sequences from this outbreak share the same lineage as those from the 2014–2016 epidemic with minimal divergence. These data strongly suggest that the 2021 epidemic was a flare-up reignited from an EBOV persistence that had lasted for six years in a human survivor of the earlier 2014–2016 epidemic, highlighting a striking long timescale of viral persistence and late reactivation.

#### 2.3.9 A new paradigm of the re-emergence of EBOV disease and transmission

As reviewed above, numerous re-emerging EBOV outbreaks have originated from a persistently infected human survivor rather than a reintroduction from a zoonotic reservoir or from another geographic location with ongoing acute EVD cases. These findings open a new perspective on EBOV transmission, shifting the old paradigm that filovirus outbreaks in humans are always the result of a zoonotic spillover from bat reservoir species or from intermediate hosts such as NHPs. Humans should now be considered an intermediate host capable of maintaining EBOV over long durations and sparking new outbreaks. Although late viral reactivation and transmission from an EVD survivor appears to be a rare event, it should be recognized that even one case can start a new outbreak with significant impact on public health, considering the high-consequence nature of EBOV infection.

It can be expected that in future outbreaks more EVD survivors will result from increased use of medical countermeasures such as vaccines and therapeutic antivirals and antibodies, which may keep severely sick EVD patients alive who would otherwise succumb. However, these survivors may be potentially at higher risk of viral persistence and its consequences: Several studies on EBOV RNA persistence in humans and animal models appear to demonstrate a link of the use of medical countermeasures before or/and during the initial acute EVD to subsequent late recrudescence, which in some cases led to onward transmission (Larsen et al., 2007; Varkey et al., 2015; Jacobs et al., 2016; Mbala-Kingebeni et al., 2021; Liu et al., 2022b; Mukadi-Bamuleka et al., 2024). While such a link remains to be confirmed with more data, it is tempting to speculate that these countermeasures confer an effective rescue from an otherwise fatal outcome, allowing and, by unknown mechanisms, facilitating the seeding and establishment of EBOV RNA persistence at certain sites with potential for viral reactivation and consequently recrudescence. Collectively, these data highlight the need for in-depth understanding of the mechanisms governing persistence, re-emergence and transmission.

#### 2.3.10 Animal models for EBOV RNA persistence

EBOV animal disease models, including NHPs, ferrets and rodents, have been generally characterized by lethal infection with an acute course, making it challenging to study EBOV persistence. However, some infected animals do survive, with or without therapeutic interventions. Findings from these animals or their archived tissues appeared to reconstitute major features of EBOV RNA persistence in humans. These confirmed the tissue sites of persistence and major associated consequences found in human clinical studies and furthermore identified new tissue sites and host cell types harboring viral RNA or/and antigens (Larsen et al., 2007; Alves et al., 2016; Zeng et al., 2017; Watson et al., 2021; Liu et al., 2022a, 2022; Worwa et al., 2022; Clancy et al., 2023; Johnson et al., 2023; Marzi et al., 2023; Beavis et al., 2025). Combining human and animal studies, the updated tissue sites of EBOV RNA persistence now include the eye, brain, semen, vaginal fluids, pregnancy-related fluids, tissues and breastmilk, male and female reproductive tracts and lung, host cell types include macrophages, epithelial cells, Sertoli cells and fibroblasts, and associated consequences include ocular inflammatory complications and vision impairment, severe/fatal meningoencephalitis, sexual transmission, inflammations in reproductive tracts, possible vertical transmission, relapse of acute EVD, pneumonia and reignited outbreaks.

## 3 MARV

While extensively implicated in the literature for EBOV, viral RNA persistence has been much less covered for other RG4 group viruses, likely due to the lack of large numbers of survivors from outbreaks as seen in EBOV. This applies to MARV, despite its belonging to the same family (*Filoviridae*, genus *Orthomarburgvirus*) and sharing similar pathogenic mechanisms as EBOV. MARV RNA persistence was found in semen, the eye and breast milk, linked to sexual transmission, orchitis, uveitis and possible mother-to-child vertical transmission (Martini and Schmidt, 1968; Gear et al., 1975; Kuming and Kokoris, 1977; Nikiforov et al., 1994; Grolla et al., 2011). NHP studies confirmed the persistence in the eye with associated uveitis, and in male and female reproductive organs with associated orchitis and potential for sexual transmission; these studies also identified Sertoli cells, epithelial cells, fibroblasts and macrophages as host cells (Coffin et al., 2018; Cooper et al., 2018).

## 4 LASV

LASV belongs to the family *Arenaviridae* as a bi-segmented, single-stranded, negative-sense RNA virus with an ambisense coding strategy (Murphy and Ly, 2021; Hastie et al., 2023). First identified in 1969 in Nigeria, the rodent-borne virus is endemic in West Africa, causing Lassa fever (LF), an acute disease characterized by hemorrhagic manifestations and multiorgan failure (Murphy and Ly, 2021; Prevost et al., 2025). The primary reservoir is the multimammate rat (*Mastomys natalensis*), though other rodents have been suggested as possible secondary hosts (Prevost et al., 2025).

In human survivors of LF, viral RNA persistence was found in semen, associated with epididymitis and potential risk of sexual transmission (McElroy et al., 2017; Thielebein et al., 2022), and CSF, associated with neurological or psychiatric sequelae (Gunther et al., 2001; Duvignaud et al., 2020; OkogBenin et al., 2020). Human survivors also demonstrated sensorineural hearing loss, vestibular symptoms and ophthalmic complications (McCormick et al., 1987; Ficenec et al., 2020; Li et al., 2020; Best et al., 2024; Wang et al., 2025). It is unknown whether these consequences were linked to viral RNA persistence. Animal infection studies have provided mechanistic insights into these post-LF symptoms. In NHP and guinea pig models, viral RNA persistence in arteries, where smooth muscle cells serve as host cells, was accompanied by systemic vasculitis, which may contribute to immune-mediated mechanisms for hearing loss and vestibular disorders (Cashman et al., 2018; Liu et al., 2019a). Additionally, in a STAT1 knockout mouse-based persistence model, viral antigen staining was observed in the inner ear, in cells of vascular-rich regions and in ganglion cells, suggesting that a direct viral effect locally may also contribute to the hearing and vestibular impairments (Yun et al., 2015; Maruyama et al., 2022; Suzuki et al., 2025).

## 5 CCHFV

### 5.1 Introduction to CCHFV

CCHFV is a bunyavirus formally belonging to the family *Bunyaviridae* but recently re-assigned into the class *Bunyaviricetes* and placed in the family *Nairoviridae* (within the order *Hareavirales*) under the updated ICTV taxonomy (ICTV, 2024b). It has a tri-segmented, single-stranded, negative-sense RNA genome. CCHFV is a tick−borne pathogen maintained in a tick–vertebrate–tick transmission cycle involving numerous wild and domestic animals. The natural reservoir hosts of CCHFV are hard-bodied ticks from the genus *Hyalomma*, and human infections occur primarily through tick exposure. Livestock such as sheep can act as intermediate or amplifying hosts facilitating onward viral transmission. The virus causes endemic and epidemic outbreaks across large regions in Asia, Europe and Africa, with emerging signs of geographic expansion. In humans, infection outcomes can range from mild or asymptomatic cases to severe acute hemorrhagic disease (CCHF) characterized by vascular dysfunction and multi-organ involvement, with case fatality rates generally reported between 5% and 30% but up to 80% depending on geographic and clinical contexts. In animals, infection typically causes a brief, transient viremia without obvious clinical signs (Li et al., 2022, 2023, 2024).

### 5.2 Potential persistence in the male reproductive tract and sexual transmission

The commonly recognized transmission routes of CCHFV include tick bites and to a lesser extent contact with infected blood, tissues or bodily fluids (Bente et al., 2013). Although sexual transmission through semen of survivors with persistent viral RNA has been reported numerous times for filoviruses, the potential involvement of male genital organs in CCHFV infection has been rarely addressed. Two cases of epididymo-orchitis, however, were documented in patients with acute CCHFV infection (Aksoy et al., 2010; Kerget et al., 2021). In both these studies, comprehensive clinical testing and evaluation ruled out etiologies other than CCHFV, including viral and bacterial pathogens known to cause epididymo-orchitis, suggesting that CCHFV may target and be present in the male reproductive tract. Consistent with this possibility, four cases of suspected sexual transmissions among couples were reported, including three from patients during their acute infection (Pshenichnaya et al., 2016) and one from a survivor after his discharge following recovery from acute infection (Ergonul and Battal, 2014). While no commonly known transmission routes such as tick bites could be identified, sexual contacts were reported in all these cases and considered a risk factor (Ergonul and Battal, 2014; Pshenichnaya et al., 2016). These findings raise the possibility that viral replication or persistence in the male reproductive tract of CCHF patients or survivors may contribute to sexual transmission. Consistently in a broader context, it was reported by other studies that for a proportion of CCHFV infections the specific mode of transmission could not be determined, and the likelihood of spreading the virus among household members through normal routine interactions other than sexual contacts appeared to be low (Ergonul and Battal, 2014). Furthermore, the possibility of reproductive tissue involvement in CCHFV infection was supported by animal model studies. Cynomolgous macaque survivors of CCHFV infection were found to harbor viral RNA persistence in the testis, where CCHFV specifically infected Sertoli cells, and develop orchitis (Smith et al., 2019). In mice immunosuppressed with an anti-IFNAR1 mAb, viral RNA persistence was detected in testes, seminal vesicles, ovary, cervix and urogenital swabs (Sorvillo et al., 2026).

### 5.3 Potential persistence in lymphoid tissues: an emerging biology of CCHFV in animals beyond an acute infection

For many decades, CCHFV infection in livestock such as sheep, cattle and goats has been described as a short−lived, transient course. According to this conventional model, at the end of a brief period of viremia, animals are assumed to recover fully with complete clearance of the virus, leaving no long−term viral footprint. However, findings from our recent experimental infection study in sheep challenge this prevailing understanding (Li et al., 2024, 2025). Our data show that CCHFV RNA remained detectable in multiple types of lymphoid tissues weeks after the acute viremia had resolved, with full−length viral genomic RNA recovered from lymph node tissues (Li et al., 2024, 2025). This directly contradicts the idea of complete viral clearance, suggesting a far more prolonged interaction between the virus and host than previously recognized.

CCHFV RNA persistence in sheep was characterized by a predominantly dormant, latency−like state, defined by several distinct molecular and virological features (Li et al., 2025). In addition to retaining full−length genomic RNA segments, infected tissues contained both viral genomic (vRNA) and antigenomic (cRNA) strands, indicating that the virus maintains the molecular infrastructure necessary for replication. Notably, the tissues lacked viral mRNA, and infectious virions could not be cultured (Li et al., 2025). Together, these features indicate a state in which viral RNA persists without continuous or extensive virion production, representing a quiescent, persistence−focused infection biology rather than a fully resolved or actively replicating state. In addition, the detection of viral proteins in rare cases suggests potentially reactivated gene expression activities out of the latent state (Li et al., 2025). Together, these findings extend the timeline of infection in livestock well beyond the acute phase and signal that animals may play a more complex and prolonged role in CCHFV ecology than previously recognized.

It is important to note that CCHFV RNA persistence in lymphoid tissues has also been detected in cynomolgous macaque and type I interferon receptor (IFNAR) knock-out mouse infection models (Hawman et al., 2019).

## 6 ANDV

### 6.1 Introduction to ANDV

ANDV is another bunyavirus that was historically classified within the family *Bunyaviridae* but, like CCHFV, has since been reassigned to the class *Bunyaviricetes*. It is now in the family *Hantaviridae* (among other hantaviruses), order *Elliovirales* (ICTV, 2024a). Despite belonging to a different viral family under the new classification, ANDV shares fundamental similarities with CCHFV in genomic structures and replication strategies (Malet et al., 2023). ANDV is endemic in Chile and Argentina and is naturally maintained in New-World rodent reservoirs, especially the long−tailed pygmy rice rat (*Oligoryzomys longicaudatus*). Humans are typically infected through inhalation of aerosolized rodent excreta. Although zoonotic transmission predominates, person−to−person spread, uncommon among hantaviruses, has been documented for ANDV. Infection in humans can lead to hantavirus cardiopulmonary syndrome (HCPS), a severe illness characterized by hemorrhagic fever combined with hyper-acute cardiopulmonary failure, with case fatality rates ranging between 25–40% (Zust et al., 2023).

### 6.2 Persistence in semen

ANDV viral RNA persistence was identified and characterized in a long-term follow up study conducted in Switzerland on an imported case of infection (Zust et al., 2023). Based on the new findings in this study, the authors raised the possibility of ANDV transmission via sexual contact, although this transmission route has not been previously documented. In the male survivor of severe HCPS, viral RNA was detectable in semen for at least 71 months (six years) post infection (while the report has not noted whether there would be further extended follow up). The RNA persistence in semen was located predominantly intracellular and did not involve DNA intermediates for integration into the host genome. Genomic analysis recovered nearly complete sequences of all the three genome segments and revealed minimal long-term viral evolution: Between two sampling time points at 247 days and 1978 days post infection (a duration of close to five years), only three point mutations and one short deletion accumulated. These data suggest limited replication activities of the persistent viral RNA reservoir, considering the error-prone nature of the RNA-dependent RNA polymerase-mediated replication of RNA viruses including hantaviruses (Ramsden et al., 2008).

### 6.3 Broader immunological evidence for viral RNA persistence across hantaviruses

Puumala virus (PUUV) is a RG3/RG2 relative of ANDV and a hantavirus endemic to Europe, causing nephropathia epidemica (NE), a relatively mild form of hemorrhagic fever with renal syndrome (HFRS, a life-threatening disease caused by other hantaviruses maintained in Old-World rodents) (Tscherne et al., 2025; ABSA, 2026; Clark et al., 2026). Recent analyses of the human antibody response to PUUV infection have revealed a prolonged and delayed maturation profile with a late emergence of broadly cross-neutralizing antibodies against multiple hantaviruses (Clark et al., 2026). A progressive shift in IgG subclass usage was characterized by the persistence of IgG1, rapid decline of IgG3, and rise of IgG4 in late convalescence several months following acute infection. These data indicate prolonged antigen exposure and continued affinity maturation of B cells well into convalescence, and imply the persistence of viral RNA encoding these protein antigens (possibly in immune-privileged sites) (Clark et al., 2026), consistent with the findings in ANDV. Furthermore, the observations in hantavirus infection in humans are also consistent with the long recognized phenomenon of lifelong, asymptomatic infection in rodent reservoir hosts (Meyer and Schmaljohn, 2000; Easterbrook and Klein, 2008; Vaheri et al., 2013; Tscherne et al., 2025), implying that elements of reservoir−like persistence of hantaviruses may manifest in human hosts as well. It should also be noted that the immune characteristics in PUUV mirror those described in human survivors of EBOV infection, where long-term viral RNA and antigen persistence has been implicated in humoral immune response evolution, with similar patterns of IgG subclass switching (Davis et al., 2019). Taken together, these observations support the concept that post-acute viral RNA and antigen persistence, sufficient to sustain long-term immune stimulation, may represent a conserved biological feature across hantaviruses and may extend to other families of RNA viruses.

## 7 Henipaviruses (HeV and NiV)

### 7.1 Introduction to henipaviruses

HeV and NiV (Li et al., 2023a) are highly pathogenic, prototypic zoonotic viruses belonging to the family *Paramyxoviridae* and genus *Henipavirus* and characterized by an unsegmented, single-stranded, negative-sense RNA genome. These viruses cause endemic and epidemic outbreaks across several regions: HeV infections have appeared sporadically in horses and a small number of humans in Australia since 1994, while NiV outbreaks include the major NiV−Malaysia (NiV−M) epizootic involving pigs and humans in Malaysia and Singapore (1998–1999), and the recurrent NiV−Bangladesh (NiV−B) outbreaks occurring almost annually in Bangladesh and India since 2001, resulting in hundreds of human infections. Their natural reservoir hosts are fruit bats, which harbor the viruses without clinical signs. Intermediate hosts, notably horses for HeV and pigs for NiV−M, play important roles in amplifying and transmitting infection to humans. For NiV−B, spillover has been linked to consumption of date palm sap, with subsequent human−to−human transmission driving outbreaks, while it remains unknown whether any intermediate hosts are involved in transmission. Acute henipavirus infection in humans typically presents as severe neurological or/and respiratory disease, underlain by a systemic vasculitis, with high case fatality rates, up to 70–100% in some outbreaks (Li et al., 2023a).

### 7.2 HeV viral RNA persistence

Some patients do survive and recover from acute henipavirus infection and long−term consequences have been observed. HeV viral RNA persistence in neurons of the brain in human survivors was associated with relapsing fatal encephalitis (O’Sullivan et al., 1997; Wong et al., 2009). A mouse model with intranasal infection route demonstrated some features of recrudescent human HeV infection, including neuron involvement and encephalitis (Dups et al., 2012), which may serve as a tool for studying aspects of recrudescence.

### 7.3 NiV viral RNA persistence

Studies across multiple NiV outbreaks provide evidence that NiV viral RNA can persist in the CNS following acute infection, and this persistence is associated with significant long−term neurological complications and the potential for late encephalitis. A follow-up study 24 months after the 1998–1999 Malaysian (NiV-M) outbreak (Tan et al., 2002) showed that 7.5% of acute encephalitis survivors had relapsed encephalitis and 3.4% of acute non-encephalitic or asymptomatic survivors had late-onset encephalitis, with a combined fatality rate of 18% among all these patients. NiV antigens were detected in the brain in neurons as well as glial and ependymal cells, suggesting a potential contribution of NiV RNA persistence (coding source of antigens) to late encephalitis (Tan et al., 2002). A survey of survivors from outbreaks in Bangladesh (NiV-B) (2003–2005) further identified a range of persistent or delayed-onset neurological abnormalities. The authors proposed that persistence of NiV RNA in the CNS, with delayed reactivation, is a plausible mechanism, mirroring patterns seen with other paramyxoviruses capable of long−term CNS persistence (Sejvar et al., 2007). Following the May 2018 outbreak in Kerala, India, NiV viral RNA was detected in the semen of a survivor beyond 16 days after viremia clearance, identifying semen as a potential site of persistence (Arunkumar et al., 2019).

An NHP model study demonstrated viral RNA persistence in grivet monkeys that survived acute NiV-M infection, providing direct experimental evidence for a mechanism underlying relapsing and late-onset encephalitis previously observed in human survivors (Liu et al., 2019b). NiV RNA and antigen were found exclusively in the brain, with no viral detection in peripheral tissues of surviving animals, indicating that the CNS serves as a unique site of viral persistence. Histopathologic examination revealed widespread encephalitis, with viral localization primarily within neurons and microglia (primary, resident macrophages) in the brainstem, cerebral cortex, and cerebellum, confirming that these cell types act as key sites of viral persistence. The study also identified vascular endothelial cells as an early target of infection during the acute phase, supporting a hematogenous route of entry into the CNS. Importantly, NiV persistence in the CNS was evident despite the clinical survival of the animals, paralleling human cases in which survivors later develop neurological recrudescence or sequelae months to years after initial illness. The findings of this study strongly suggest that viral persistence in the brain is a plausible cause of relapsing and late-onset encephalitis in human survivors, and that understanding this process is essential for unraveling the pathogenesis of late henipavirus disease. One major unanswered question is in what form NiV persists. Although viral RNA and antigens were detected in the brain, virus isolation was not attempted.

## 8 TBEV

### 8.1 Introduction to TBEV

TBEV is a flavivirus (family *Flaviviridae*), with an unsegmented, single−stranded, positive−sense RNA genome (Ruzek et al., 2019; Pustijanac et al., 2023). It is endemic across a vast geographic range, from central, northern and eastern Europe to northern and eastern Asia, with a concerning trend of expansion into traditionally non-endemic regions (Lindquist and Vapalahti, 2008; CDC, 2025). The Baltic and Central European countries have the highest incidence of TBEV infection (Lindquist and Vapalahti, 2008; Pustijanac et al., 2023). There are three main subtypes of TBEV (European, Siberian and Far Eastern) and two other types (Baikalian and Himalayan), which emerged more recently (Ruzek et al., 2019). The circulation of TBEV involves ticks from the genus *Ixodes* as the primary natural reservoir hosts and small mammals as vertebrate reservoir hosts. Humans are incidental hosts and become infected mainly via tick bites, while consumption of unpasteurized dairy products was identified as an additional, food-borne transmission route (Ruzek et al., 2019). Acute TBEV infection can range from mild, nonspecific symptoms to severe neurological disease, including meningitis, encephalitis or myelitis (Vilibic-Cavlek et al., 2024). Case fatality rates vary significantly by subtype and region, ranging from less than 2% for the European subtype up to 8% for the Siberian subtype and 20–40% for the Far Eastern type (Vilibic-Cavlek et al., 2024; Kelly et al., 2025). Although TBEV is often considered for RG3, the Far Eastern type, with a much higher fatality rate, is particularly classified into RG4 by countries like the United States (CDC/NIH), Switzerland and United Kingdom. It should be noted that in Canada all TBEV strains, regardless of subtypes, are strictly classified as RG4 (PHAC, 2023). In addition, it has also been recommended in the United States that laboratory work with TBEV (generally, regardless of subtypes) be restricted to biosafety level 4 facilities and practices (Hills et al., 2023).

### 8.2 Possible decade(s)-long viral RNA persistence linked to late fatal neurologic disease

A large proportion of survivors of acute TBEV infection develop long-term neurological complications, although the numbers vary substantially across studies and depend on multiple viral and host factors such as the TBEV subtype and host age (Lindquist and Vapalahti, 2008; Halsby et al., 2024, 2025; Kelly et al., 2025). Long-term sequelae of TBEV infection is often believed to arise from immune−mediated injury, neuroinflammation and structural CNS damage sustained during the acute encephalitic phase (Kelly et al., 2025), due to the lack of mechanistic investigations into the potential contribution of viral persistence in human clinical studies. However, animal model studies in NHPs, hamsters and wild rodents have demonstrated viral RNA persistence in CNS (and lymphoid tissues) associated with late encephalitis (Frolova et al., 1982a, 1982; Malenko et al., 1982; Frolova and Pogodina, 1984; Malenko et al., 1984; Frolova et al., 1987; Malenko and Pogodina, 1989; Tonteri et al., 2011, 2013). Indeed, viral RNA persistence may be involved in human cases of fatal late-onset (Gritsun et al., 2003) and relapsing (Volok et al., 2022) neurologic disease.

In the first case (Gritsun et al., 2003), a patient developed a fatal, progressive encephalitis with a 2−year period of gradual deterioration preceding death, where a TBEV strain of the Siberian subtype was isolated. Epidemiological investigation tracked the infection back to a tick bite 10 years before the disease onset, while no other possible source of transmission was identified. These observations point to the possibility of TBEV persistence within the host for a record−setting duration of 10 years.

In the second case (Volok et al., 2022), a patient was initially diagnosed with acute TBEV infection with moderate motor neuron disease symptoms after being bitten by an infected tick on his neck in Siberia (Irkutsk region). Nearly 35 years later (in Moscow region), he relapsed with a worsened and fatal motor neuron disease (amyotrophic lateral sclerosis-like syndrome). TBEV infection status during his disease stages was confirmed by the detection of antibodies to different TBEV proteins in the serum and CSF. While the report did not show detectable TBEV RNA, the authors cited that even with the most sensitive diagnostic assays, TBEV RNA is rarely detectable in CSF, and its absence does not rule out the presence of viral activity (Schwaiger and Cassinotti, 2003), and in addition, the detection of anti−NS1 antibodies in the CSF of the patient was consistent with the possibility of ongoing or recent reactivation in the CNS rather than distant past infection (Ruzek et al., 2019). This case documents by far the longest reported interval, 35 years, between acute TBE infection and symptomatic relapse. Convergent epidemiological and clinical evidence suggests that the patient’s fatal neurological disease resulted from true 35-year persistence of TBEV followed by late viral reactivation. The late disease was essentially unlikely to have resulted from a recent re-infection. To the current knowledge, no cases of TBEV reinfection have been documented in people who survived the clinically manifest disease, and here there was no evidence of new tick bites or other potential exposure events that could explain a re-infection. Furthermore, despite the vast diversity and variability of neurological manifestations generally among TBEV infections (Volok et al., 2022), the clinical presentation during the relapse here closely mirrored that of the initial acute infection, with the same anatomical areas (such as the neck, shoulders, and throat) being affected, strongly indicating a recrudescence of the prior infection. Collectively, these findings demonstrate the longest documented latency period to date for TBEV or non-retro RNA viruses as a whole and illustrate the capacity of persistent viral RNA to remain dormant for decades but later cause devastating disease.

### 8.3 Inducible reactivation

Several early studies published in Russian described a hamster model of TBEV persistence in which latent infection could be experimentally reactivated by certain immunosuppressive chemotherapeutics or hormones, or antibiotics. Reactivation was observed with chemotherapeutics cyclophosphamide (cyclophosphane) (Frolova et al., 1982b) and vincristine (Frolova and Pogodina, 1984), immunosuppressive hormones prednisolone and adrenaline (Frolova and Pogodina, 1984) and antibiotic streptomycin (Malenko et al., 1984). In a subsequent study searching for antibiotics that do not stimulate reactivation of persistent TBEV, levomycetin showed no activating effect, while kanamycin and florimycin exhibited only weak activation (Malenko and Pogodina, 1989). In the recently documented case of 35−year TBEV persistence, disease recrudescence occurred following an unusually long 120−km bicycle ride, suggesting that physical stress may trigger reactivation (Volok et al., 2022). These observations highlight an inducible nature of viral reactivation from long-term persistence.

### 8.4 Viral RNA persistence in a broader flavivirus context: lessons from Zika and yellow fever viruses

ZIKV and YFV are mosquito-borne flaviviruses, and RG2 and RG3 relatives of TBEV, respectively (Musso and Gubler, 2016; Douam and Ploss, 2018). ZIKV represents a well−studied example of post−acute viral RNA persistence in humans, as ZIKV RNA has been repeatedly detected in semen for prolonged periods, frequently extending for several months following an acute infection (Harrower et al., 2016; Nicastri et al., 2016; Atkinson et al., 2017; Paz-Bailey et al., 2018). Importantly, ZIKV persistence in semen has proven clinically and epidemiologically relevant, as multiple well−documented cases have confirmed sexual transmission of ZIKV (Harrower et al., 2016; Turmel et al., 2016; Russell et al., 2017). Extending the findings in humans, an animal model study reported ZIKV RNA persistence in neuronal, lymphoid and joint/muscle tissues as well as the male and female reproductive tracts, providing mechanistic insights into ZIKV neuropathogenesis and sexual transmission (Hirsch et al., 2017). YFV infection is characterized by a biphasic illness often progressing to a severe “intoxication” phase after an initial period of remission (Thomazella et al., 2026). Historical experimental investigation in NHPs reported YFV persistence in the brain well beyond an initial acute stage of infection (Penna and Bittencourt, 1943). Recent studies have provided renewed evidence in naturally infected NHPs demonstrating the presence of YFV RNA and antigen in the brain (Cunha et al., 2019; Oliveira et al., 2025). Together, these observations from ZIKV and YFV suggest that post−acute viral RNA persistence may be a shared feature of flaviviruses rather than an exception unique to TBEV. Recognition of this broader pattern may help reframe concepts of TBEV pathogenesis and motivate more systematic investigation of flavivirus persistence across both human and animal hosts.

## 9 A mechanistic perspective on the persistence of zoonotic non-retroviral RNA viruses: the latency hypothesis

Little is known about the mechanisms by which zoonotic non-retroviral RNA viruses persist between outbreaks. Across outbreaks sparked by EBOV RNA persistence in humans, a phenomenon described as “a reduced evolutionary rate” has been consistently observed: no or few mutations accumulate during prolonged persistence, for months and years (Rodriguez et al., 1999; Blackley et al., 2016; Diallo et al., 2016; Jacobs et al., 2016; Liu et al., 2019c; Keita et al., 2021; Mbala-Kingebeni et al., 2021). These mutations are by far fewer than would be expected if the virus had continued replicating during sustained human-to-human transmission given the EBOV mutation rates during replication. Similarly, reduced evolutionary rate also occurs in persistent RNA of ANDV (Zust et al., 2023). It is a mystery how these viruses persist in a host for long periods of time without mutating. However, this minimal genetic divergence reveals the possibility that the viral RNA remains predominantly in a latent, non-replicating state during the long periods of persistence. It is also tempting to speculate that viral RNA replication and thus mutation only occur during rare events of viral reactivation. Similarly to that observed in humans, a recent phylogenetic study on the natural history and reservoir dynamics associated with zoonotic spillover events suggested that EBOV can persist in a dormant state in its natural reservoir for extended durations (McCrone et al., 2025). In these contexts, we propose that latency may serve as an evolutionary mechanism contributing to viral persistence throughout the natural history of zoonotic non-retroviral RNA viruses. While the primary evidence motivating this hypothesis comes from insights gained with RG4 viruses such as EBOV, ANDV and TBEV, we speculate that similar mechanisms may extend to a broader range of zoonotic non−retroviral RNA viruses, albeit potentially to varying degrees. It should be emphasized that, although latency is a well−established mechanism underlying viral persistence in DNA viruses (Han, 2025) and retroviral RNA viruses (Dufour et al., 2020), its existence and relevance remain undefined in non-retroviral RNA viruses, as these viruses are traditionally considered to lack latency programs (Griffin, 2022). Listed below are the key principles of the Latency Hypothesis that we propose for zoonotic non-retroviral RNA viruses.

- Non-retroviral RNA can maintain long durations of latency (quiescence) without gene expression or viral replication in a natural reservoir host, as an evolutionary mechanism to sustain long-term viral survival. This also allows the virus to lie dormant for years between outbreaks, explaining the sporadic nature of human outbreaks.
- Rare, occasional or intermittent reactivation from latency to replication contributes to the maintenance and renewal of persistent cellular reservoir within the host. It also has the potential for viral transmission to immunologically-naive animals, which leads to the establishment of a new cohort of persistently infected hosts, as well as transmission to humans igniting outbreaks. Genetic changes occur only during reactivated replication, explaining slow evolution through persistence. Reactivation may be triggered by changes in host physiology and immune modulation, making the cellular environment more favorable for viral gene expression or replication.
- The dynamic cycles of dormant and active infections are a fundamental part of non-retroviral survival strategy and the regulation of latency by internal and external signals governs when and where reactivation, transmission and outbreak emerge.
- The frequency of viral reactivation from latency may vary substantially across different viruses. EBOV may represent an extreme case, with reactivation potentially not occurring for several years. This may have at least in part contributed to the unsolved mystery of natural hosts for EBOV, which have evaded identification since 1970s. Due to the rarity of viral reactivation events, the isolation of live virus is anticipated to be highly opportunistic in nature and requires sampling large numbers of animals at the right timing and location. It should be noted that it is likely several factors must align for a fortuitous success. Recent experimental infection studies have identified Angolan free-tailed bats as a promising candidate reservoir for EBOV (Riesle-Sbarbaro et al., 2024). They should be included in future surveys alongside other potential reservoir hosts.
- In reservoir species, co−evolution may favor tolerance mechanisms with virus–host immune system interactions finely equilibrated that allow long-term viral latency and maintenance without apparent pathology.
- Latency with potential for reactivation is not unique to persistence in reservoir hosts but can be exploited to achieve persistence in spillover hosts. In non-natural hosts, shifts in the execution of viral or host tolerance mechanisms can lead to disease following reactivation from latency.
- The hypothesized viral RNA persistence and reactivation cycles appear to be consistent with long-lasting or recurrent host immune responses attesting to stimulations by antigens resulting from renewed replications (Fabiyi and Tomori, 1975; Natesan et al., 2016; Hawman et al., 2019; Yadav et al., 2019; Wiedemann et al., 2020; Adaken et al., 2021; Thom et al., 2021; Zust et al., 2023; Khurana et al., 2024; Vasmehjani et al., 2024; Cohen et al., 2025; Mihalakakos et al., 2025; Ong et al., 2025; Ibrahim et al., 2026).
- These principles provide a rough sketch of latency potentially serving as a central component in the biology of viral RNA persistence. With the field only beginning to be explored, the molecular details of the mechanisms behind latency remain to be filled in. Non-retroviral RNA viruses do not possess a reverse transcriptase that would allow their RNA to be converted into DNA and integrated into the host genome. At present, no mechanisms have been clearly identified that sustain viral RNA persistence independent of a DNA phase in the absence of ongoing replication. Thus, a primary unknown is in what form persistent viral RNA exists. As consistently found across many studies, the successful isolation of infectious virus has proven to be rare, strongly suggesting the general absence of whole, infection-competent visions during latency. It is rather likely that latency is maintained in the form of large molecular ribonucleoprotein (RNP) complexes harboring an intact viral genome with coding capacity to fulfill the whole viral replication process upon reactivation. Indeed, CCHFV RNA persistence in sheep tissues is characterized by full-length viral genomic sequences even when no culturable virus could be recovered (Li et al., 2024, 2025).

## 10 Concluding remarks

Traditionally underappreciated, an emerging biology of post-acute non-retroviral RNA persistence is unfolding. Accumulating evidence indicates that viral RNA persistence is not merely a molecular footprint of infection but represents a functional biological process that can lead to long-term sequelae, recrudescence and viral transmission. Although a rare event, reactivation of zoonotic RG4 RNA viruses from latent viral RNA reservoir persisting in natural hosts or human survivors pose catastrophic consequences to individual and public health. The viral RNA reservoir in a host can last for months, years and possibly decades, and the longest duration limit is unknown. Cycles of reactivation and subsequent viral transmission may fuel long-term viral maintenance and expansion in natural host populations and human survivors. The mechanistic determinants of RG4 viral persistence remain unexplored but their elucidation is essential for developing therapeutic strategies to achieve complete clearance of the latent RNA reservoir and mitigating the long-term public health risks posed by these high-consequence pathogens.

## Supplementary material

Supplementary Table 1 Persistent RNA in RG4 zoonotic viruses and associated clinical and public health implications. An excel file containing the table and references.
